# Cosmetic tail docking: an overview of abuse and report of an interesting case

**DOI:** 10.1186/s12917-016-0666-z

**Published:** 2016-02-29

**Authors:** Oghenemega David Eyarefe, Cecilia O. Oguntoye

**Affiliations:** Department of Veterinary Surgery and Reproduction, Faculty of Veterinary Medicine, University of Ibadan, Ibadan, Nigeria

**Keywords:** Dog, Tail docking, Abuse, Rubber rings, Animal welfare

## Abstract

**Background:**

This paper presents an overview of the global controversies surrounding cosmetic tail docking in puppies, some observed inconsistent practices among dog breeders and Veterinarians in West Africa, and the need for the African Veterinary Profession to take a decisive position on the cosmetic docking procedure.

**Case presentation:**

An interesting report of observed complications associated with cosmetic tail docking in a 3 week old male Boerboel is reported alongside the management of the ensuing complications.

**Conclusion:**

This paper highlights the still prevalent practice of cosmetic tail docking and seeks to enlighten clinicians towards stemming its abuse in Africa.

## Background

Tail docking is the amputation of a part or all of an animal’s tail [1]. In puppies, it is usually performed between day three to five of life or at 3 months under general anaesthesia by surgical amputation with a scalpel [2]. Some breeders, however, perform tail docking without anaesthesia by the application of tight rubber rings around the tail which serves to occlude vessels caudal to the rubber ring, resulting in ischemic necrosis and sloughing of the tail [1, 3]. Tail docking in animals, especially dogs, remains a controversial subject among veterinarians, animal breeders, pet owners and animal welfare groups in many countries of Europe and the United States of America (USA) [1, 4, 5]. The procedure has been banned in the United Kingdom with exceptional provision made for therapeutic and prophylactic tail docking in certified working dogs [6]. Cosmetic tail docking is gradually becoming an issue in Africa with South Africa leading in the ban against the procedure [7] while other African countries are yet to have a legislative position on the procedure.

Tail docking in many dog breeds is an established custom believed to have been introduced some 2000 years ago [1]. In recent times, dogs’ tails are supposedly docked to conform to breed standards, prevent tail injuries, and to potentially reduce the accumulation of fecal materials around the tail area of dogs with excessive coat [1, 8]. Docking dogs to prevent tail injuries has, however, been controverted by many recent studies [9, 10]. In a study conducted in Great Britain, to assess the risk of tail injury and associated risk factors, as well as, to allow objective assessment of the frequency of tail injury and risk factors associated with them [9]; the overall risk of tail injuries was low. The weighted risk was 0.23 % per year, with working-dogs being 0.29 % and non-working dogs 0.19 % [9]. The study concluded that, although docking appears to be protective against injury, over 500 dogs would need to be docked in order to prevent one tail injury [9]. In another recent study to assess the nature of canine tail injury in New Zealand [10], it was concluded that tail injuries are rarely observed in Veterinary clinics, and docking a risk factor in traditionally docked breeds [10]. Tail docking is associated with severe acute pain which often causes behavioural distress in puppies [11] especially when performed without anaesthesia or analgesia, especially as with rubber ring. Chronic pain arising from tail stump infections and neuromas have also been reported [12–14], and elucidated with pain studies in other species [15]. Chronic health challenges such as faecal incontinence, atrophy of pelvic muscles [5], frequent tail damage [9, 16, 17], impaired locomotory and communication defects have also been reported and confirmed through previous studies [4, 5]. These complications, and lack of dog’s benefit from the procedure have raised strong oppositions from Veterinary associations and animal welfare groups [3, 18, 19] resulting in the ban of non-therapeutic animal docking in many European countries, Australia and South Africa [3, 7, 20–23].

The current influx of traditionally docked breeds into major countries of Africa including Nigeria has heightened the non-therapeutic dog tail docking practice [7], with non-compliance to docking time for puppies [7], abuse of the rubber band docking method, indiscriminate docking of dog breeds and non-cognisance of the required number of residual coccygeal vertebrae in line with breed standards (Authors’ unpublished observations). These have resulted in an upsurge of post-docking complications and animal suffering. This paper, which is the first of its kind from Nigeria, reports one of such tail docking abuses, and the ensuing complications as evidence of cruelty to companion animal species, and a call for a strong legislation towards the ban of cosmetic tail docking in all African Countries .

## Case presentation

**Fig. 1:**
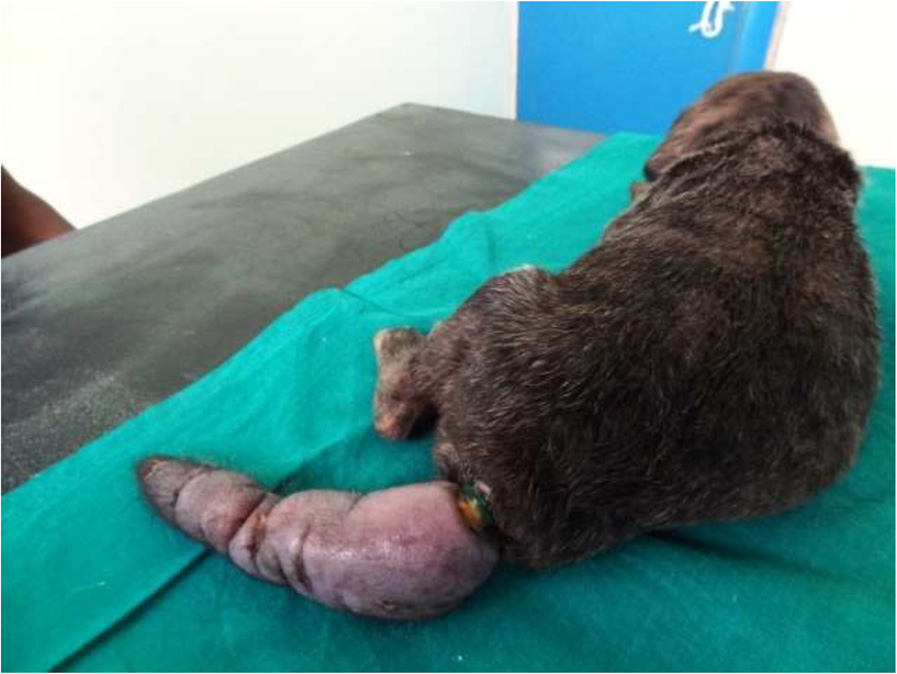
Initial presentation: a swollen, ischaemic, necrotic tail. Legend: The initial picture of the tail is shown at the point of presentation. At this point, the tail has failed to slough off after 2 weeks of rubber ring application. The tail is swollen and necrotic and painful to the touch. When the pup attempts to move, it does so with the tail being dragged on the floor behind it

**Fig. 2:**
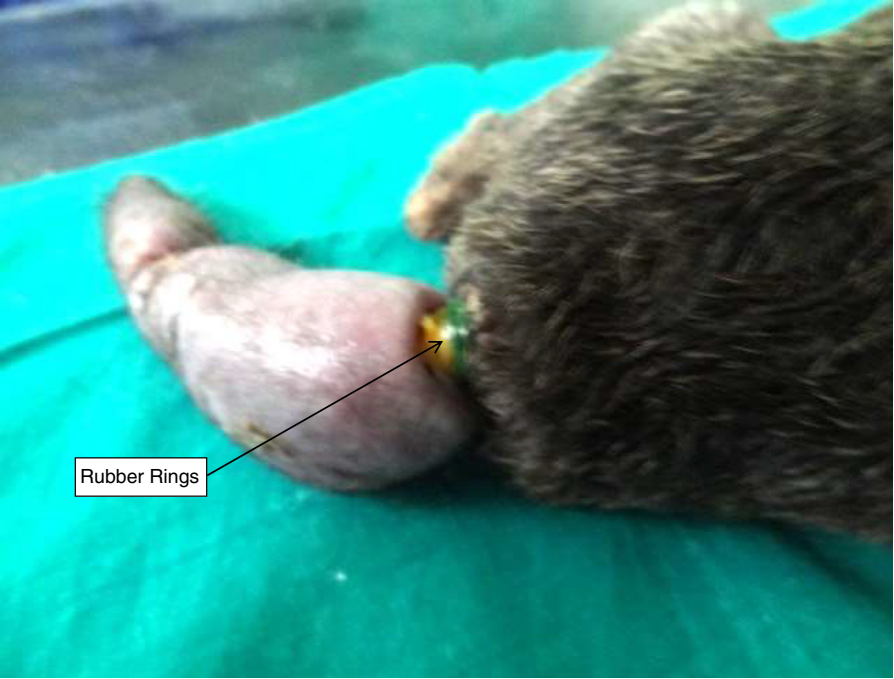
Puppy’s tail with rubber ring attachment. Legend: The point of application of the rubber rings is highlighted, just at the root of the tail where it joins to the hip. Several rings of rubber bands are clearly visible

A 3 week and 2 day old male Boerboel was presented at the Surgery Unit of the Veterinary Teaching Hospital of the University of Ibadan, Ibadan, Nigeria, with a severely swollen, gangrenous tail which, according to the owner, occurred following an attempt to amputate the tail with a rubber ring tied tightly on the tail two weeks previously (Figs. 1 and 2). This puppy’s tail did not slough off; though his six other litter mates did about 7 days following the placement of the band. The puppy was severely distressed with pain at presentation; evidenced by continual vocalisation. Owner also complained of loss of appetite and un-thriftiness among its litter mates.

### Physical examination

The puppy weighed 2 kg. Its rectal temperature was normal (37.6 °C) but other physiological parameters were slightly elevated although within normal range. The point of rubber band application was septic (Fig. 2), and the rubber rings were on the second coccygeal vertebrae.

### Surgical treatment

**Fig. 3:**
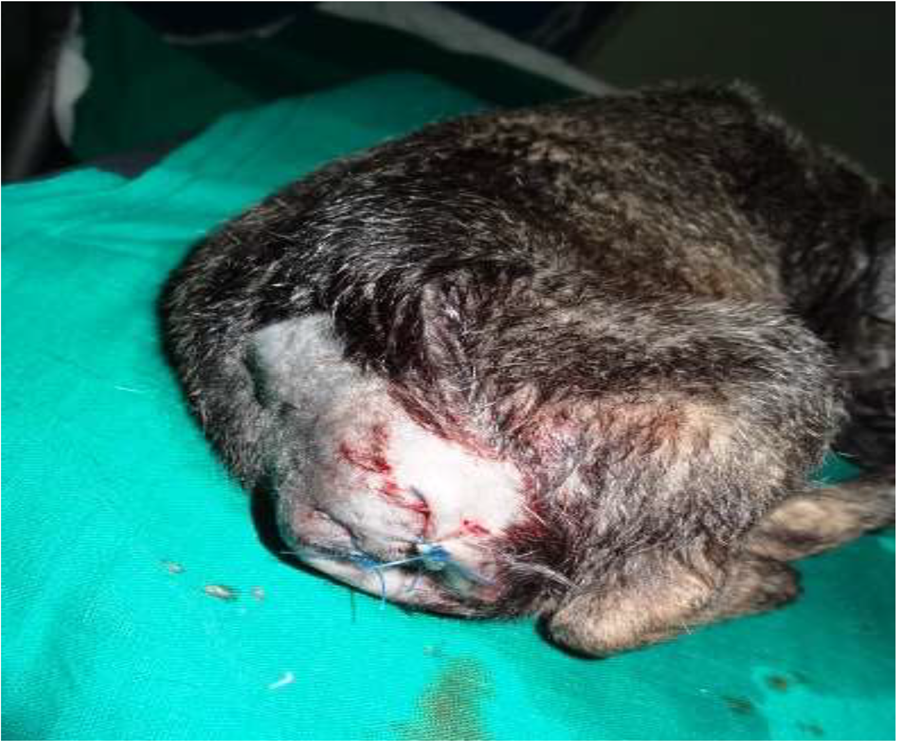
Tail stump after surgical therapeutic docking. Legend: After therapeutic excision of the affected tail, the stump is pictured

**Fig. 4:**
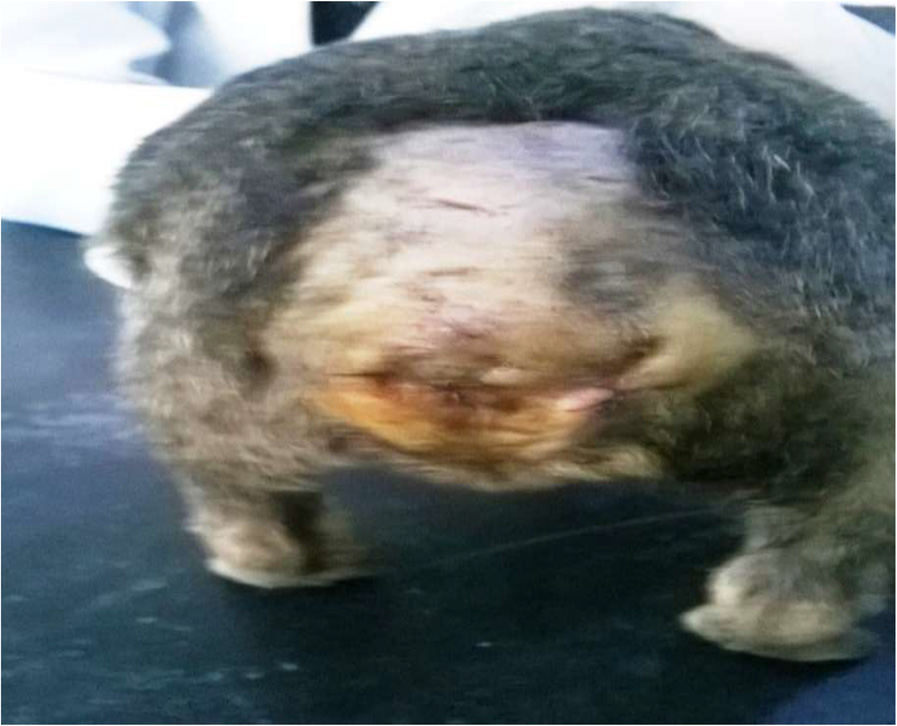
Tail region after a removal of sutures. Legend: A picture is shown after the sutures are removed. The stump is healing nicely as expected, with uneventful cosmetic appearance

Following 2 intranasal drops of Ketamine hydrochloride (0.1 mg/100 g) which sedated the patient, as previously described [2], lumbosacral epidural nerve block was done with 2 % lignocaine (Glocain, Vital Care Limited, India) at a dose rate of 1 ml/6 kg body weight [24]. Docking was at the root of the tail and incision closed with cruciate suture pattern using size 1-0 nylon sutures (Fig. 3). Healing was uneventful, sutures were removed (Fig. 4) and puppy grew rapidly to equal litter mates’ weight within 2 weeks.

## Discussion

Cosmetic tail docking remains controversial for acute and chronic pain associated with the procedure [3]. This is more severe in puppies due to incomplete myelination of their nerve sheet which make them more sensitive to pain than adults [13]. As previously reported [3], the tail is a complex anatomical structure comprising of ligament, muscles, and tendon, well innervated and vascularised. It is absolutely cruel when the pain threshold from the time of fixing the rubber ring is imagined [11], as each puppy struggles through the period of tightened rubber band leading to ischaemic necrosis and sloughing of the tail. Although rubber ring method is conventional for prophylactic tail docking in lambs; aside gas heated hot knife and sharp knife [25], the method is also reportedly associated with intense pain [26] and high cortisol response [27, 28], abnormal postural display [29] irrespective of docking age [30]. There is also a prolonged average period of 28 days before the tail sloughs off [31]. This may explain why the rubber ring method though tolerated in lambs may not be suitable for prophylactic tail docking in dogs. Besides, a more intense pain and sloughing prolongation have been reported when docking rings are placed on the vertebrae instead of in-between vertebrae [32]; a position which may be difficult to ascertain by un-trained persons. The phylogenic differences between the ovine and the canine species (tail muscle size, length, rate of coccygeal osteogenicity) may also explain while the rubber ring method may be less suitable in canine species [33]. Attempt at docking this puppy was made at the ninth day of life, instead of between day three to five as contained in literature [2]. The development of coccygeal cartilage to bone may have also contributed to the docking failure. Besides, docking of this puppy beyond the time suggested in literature shows the desperation of breeders to dock their dogs’ tail without consideration of inherent complications associated with their wrong actions. This is more pronounced in poor resource setting of many African countries where money is a prime factor, and docking is done to enhance sales of puppies [7]. The use of intranasal sedation/ anaesthesia with ketamine hydrochloride was reported in literature for puppy docking [2]. The process enhanced chemical restraint of puppy and eased administration of epidural nerve block to provide intra-operative and postoperative analgesia [34].

## Conclusions

Cosmetic tail docking is cruel to puppies, and other species, especially when done without anaesthesia. Veterinary and Animal Welfare Associations in African countries should therefore move a legislative process that could lead to a ban of the procedure.
